# Structural developmental psychology and health promotion in the third age

**DOI:** 10.1093/heapro/daw104

**Published:** 2017-01-11

**Authors:** Lars Bauger, Rob Bongaardt

**Affiliations:** 1 Department of Nursing and Health Sciences, Faculty of Health and Social Sciences, University College of Southeast Norway, Porsgrunn, Norway; 2 Department of Health, Social and Welfare Studies, Faculty of Health and Social Sciences, University College of Southeast Norway, Porsgrunn, Norway

**Keywords:** health promotion programs, quality of life, qualitative methods, older people

## Abstract

In response to the ever-increasing longevity in Western societies, old age has been divided into two different periods, labelled the third and fourth age. Where the third age, with its onset at retirement, mostly involves positive aspects of growing old, the fourth age involves functional decline and increased morbidity. This article focuses on the entry to the third age and its potential for health promotion initiatives. Well-being is an important factor to emphasize in such health promotion, and this article views the lifestyle of third agers as essential for their well-being. The structural developmental theory of Robert Kegan delineates how a person’s way of knowing develops throughout the life course. This theory is an untapped and salient perspective for health promotion initiatives in the third age. This article outlines Kegan’s approach as a tool for developing psychologically spacious health promotion, and suggests future directions for research on the topic.

## INTRODUCTION

Retiring from work is a major transition in life and in many countries. It is the social marker of entering into old age (Kloep and Hendry, 2006). The conception of old age altered dramatically during the late 20th century as people lived increasingly longer. As one consequence of this, researchers now distinguish between the ‘third age’ and ‘fourth age’ (Baltes, 1997; Baltes and Smith, 2003; Laslett, 1996). In gerontology the last stage of a person’s life is often called the fourth age (Koss and Ekerdt, 2016), which is a period characterized by functional decline and an increased dependency. The third age, with its onset in retirement, is seen as a period of relatively good health with the potential of active social engagement forming a solid base for healthy ageing (Robinson, 2013). Even though the third age has a positive ring to it, it may come with some challenges that are specific for this period of life. Retirement itself, whether it comes voluntarily or, as may happen, involuntarily, may be experienced as troubling (Daatland and Solem, 1995) and can have a negative effect on the well-being of the retiree (Wang, 2007). Studies of retirement effects on the person’s well-being have demonstrated that between 9-25% experience negative effects to their well-being after retirement (Wang, 2007; Pinquart and Schindler, 2007). In their recent review, Wang and Shi (2014) highlighted different factors pre, during and post retirement that affected the well-being of the retiree. The negative factors were ill health, involuntary retirement, a concern with the maintenance of social status and contacts, and strongly identifying with one’s work role (Wang and Shi, 2014). Health promotion may help retirees to find a suitable place in society and improve well-being in spite of these negative factors. In addition, health promotion will prolong this third age period and as a consequence likely compress morbidity during the fourth age period (Whitehead, 2011). Whitehead (2011) also suggests that during the fourth age, persons may draw on existential forces to overcome adversity, forces that are built up during earlier stages of life, including the third age. Health promotion is apt to endorse such existential forces. In other words, health promotion during the third age may postpone the onset of the fourth age, make it shorter and more endurable once the person faces adversity.

Health promotion typically targets large populations and may be unable to address individual differences. The third age population, however, is characterized by an immense heterogeneity (George, 2011; Wang, 2007), and many third agers have acquired a unique professional competence, a specific way of living, and a network that intertwines two or three generations of family and friends. Ideally, health promotion should be individually tailored to the needs of each third ager. However, the group’s heterogeneity renders that unfeasible. In this article we outline a feasible approach to shaping health promotion, directed at the intermediate range between a large population and the unique individual. We do so by introducing the structural developmental theory to the healthy ageing discourse and linking this to the policy making and practice of healthy lifestyle promotion. The specific purpose of this article, then, is to outline a structural developmental approach to the field of health promotion that targets the well-being of third agers. Before presenting the structure of the rest of this article, we will first delineate some central concepts.

The structural developmental theory focuses on consecutive stages of mental structures in a person’s life. Such a theory is perhaps best introduced by contrasting it to phase theories of life course development that emphasize normative phases of life, such as birth, childhood, education, young adulthood, marriage, parenthood, working life and retirement (e.g. Erikson, 1980). Whereas the phase developmental theory focuses on the content of age-dependent periods of life, structural developmental stage theory underscores how this content is put into perspective by the person – i.e. the extent to which one takes responsibility for the unfolding of events, and, ultimately, how the story of one’s life is told at any particular moment in time. The development of these perspectives is referred to as the development or growth of complexity of mind (Kegan, 1994).

The field of health promotion often refers to the life style concept. However, definitions of the lifestyle concept abound. We assume that lifestyle is made of the fabric of a person’s attitudes, manners, behaviours and practices, which are all woven into a Gestalt (Cockerham, 2005; Elstad, 2000). In our view, a person’s complexity of mind underpins his or her lifestyle. We thereby emphasize coherence in what are often presented as separate lifestyle ‘factors’, such as smoking, diet, exercise, etc. (cf. Veal, 1993). Furthermore, lifestyle and well-being can be seen as reciprocally related – well-being is embedded in lifestyle and takes shape through it. Well-being is a heavily debated topic within health psychology and we are not advocating for any of its schools of thought. In this article, we take a broad perspective and focus on the subjective experience of the phenomenon. Nevertheless, our use of well-being is in line with how Huppert (2009 p.137) defines psychological well-being, i.e. ‘the combination of feeling good and functioning effectively’. Feeling good, then, is not just concerned with happiness and contentment but additional emotions such as ‘interest, engagement, confidence and affection’ (2009, p. 138), whereas functioning effectively captures ‘the development of one’s potential, having some control over one’s life, having a sense of purpose (e.g. working towards valued goals), and experiencing positive relationships’ (2009, p. 138).

The structure of this article is as follows. We first review and present the key concepts of our article; the third age, health promotion and lifestyle. Then we summarize Kegan’s theory of structural development of the mind. After that, we present the design of a study that addresses the experience of well-being premised on complexity of mind, and, finally, discuss the logical implications of a psychological developmental approach to tailoring health promotion for third agers.

## HEALTH PROMOTION AT THE ONSET OF THE THIRD AGE

A positive perspective on the third age is well captured by the gerontology term ‘successful ageing’. The term gained popularity during the last decades of the 20th century (Baltes and Smith, 2003). It was introduced by Rowe and Kahn (1987) who reacted to the tendency in gerontology to distinguish only between older people with disease or disability and those without such conditions. They introduced successful ageing as a positive concept in order to address high cognitive and physical functioning and an active engagement with life, in addition to a low probability of disease and disability. In the newfound optimism in the field of gerontology, the perception of ageing changed from a passive experience to a process of active engagement and participation (Baltes and Baltes, 1990).

This more optimistic perspective on ageing has influenced political discourse (Villar, 2012), as witnessed by the introduction of the term ‘active ageing’ by the World Health Organization (2002). The WHO defines active ageing as ‘the process of optimizing opportunities for health, participation and security in order to enhance quality of life as people age’ (2002, p. 6). The WHO policy is to promote active ageing as a way to address societal and economic challenges stemming from an ageing population as well as individual challenges associated with getting older (World Health Organization, 2002). Here the focus is on adding ‘more life to years, not just years to life’ (Vaillant, 2004, p. 561), which is a hallmark of health promotion in the third age. Wilson and Palha (2007) argue that health promotion during this transitional period will not only assist in maintaining existing health but could also improve health and well-being simply because this is a period when one has more time to attend to health-related needs than when one was working. The third age is a period where one is left more to one’s own devices with few established social structures and socially defined roles (Freund *et al.*, 2009). People are often more free to do what they want, but those who do not know or have not planned for what to do with this new freedom could easily become ‘passive and couch ridden’ (Solem, 2012, p. 88; our translation).

It is evident that retirement is seen as an important period for health promotion efforts. However, retirement-specific research on health promotion is still in its early stages. Reviewing the research, Wilson and Palha (2007) identified 20 studies on the topic. Their content analysis of these studies revealed four major themes in the research on health promotion at the onset of the third age, i.e. retirement: (1) the considerable effect of retirement and the need to support positive retirement, (2) the identification and overcoming of barriers to health promotion at retirement, (3) the best methods to promote and sustain healthy lifestyle changes among retirees and (4) the short and long-term benefits of health promotion at retirement (Wilson and Palha, 2007). Given the aim of the present article, we will elaborate on theme (3), which links successful ageing to the promotion of healthy lifestyles.

We emphasized above that the Gestalt of a person’s attitudes, manners, behaviours and practices can be seen as his or her lifestyle. A lifestyle approach to health promotion builds on the assumption that the individual can amend this lifestyle (Elstad, 2000; Nutbeam, 1998). Although studies show that adopting a healthy lifestyle may be beneficial for healthy ageing, the literature reports some difficulty in promoting a healthy lifestyle through interventions (Zhang *et al.*, 2013). The main focus has been restricted to financial planning (Osborne, 2011), whereas psychological or social changes that might occur after retirement have received hardly any attention (Kloep and Hendry, 2006). Health promotion initiatives usually communicate messages about healthy lifestyles to a large target population through health education booklets or pamphlets. Kreuter *et al.* (1999) have criticized this way of promoting health for its ‘one-size-fits-all’ approach, with little consideration of individual needs and personal relevance. In response to this criticism, there has been a growing interest in tailoring interventions to different individual users and user groups (Davis, 2008; Orji and Mandryk, 2014). We share this interest and wish to contribute. Our contribution to the development of tailor-made methods to promote and sustain healthy lifestyle changes among retirees is based on structural developmental theory, which we describe in the following section.

## STRUCTURAL DEVELOPMENTAL THEORY

Neo-Piagetian psychologist Robert Kegan developed a structural developmental theory (1982, 1994) which proposes that individuals interpret and make meaning of their world in qualitatively different ways. These ways of meaning-making develop throughout the life course along an invariant path whereby more complex ways of meaning-making build upon and transform earlier ways of meaning-making. The ways of meaning-making are termed structures or orders of mind. Kegan (1982) has described three orders of mind that capture most of the adult population. He refers to these orders as the socialized, the self-authoring, and the self-transforming mind (Kegan, 1994). Each order captures what an individual can take as an object – can see ‘in front of’ him or her – and what an individual is subject to – is part of and thereby lacks a perspective on.

Individuals who have developed a socialized order of mind can think in abstract terms and have the capacity to internalize the meaning systems of others, such as family values, social values, professional culture, etc. They have the ability to subordinate their own desires and be guided by the norms and standards in the ideologies, institutions or people that are most important to them (Fitzgerald and Berger, 2002). At this order of mind, one easily sees beyond one’s own needs and can adopt a larger picture, in which one is part of a socially defined reality. Even though one has the capacity to internalize others’ points of view, one is embedded in these points of view and is essentially dependent on them. That is to say, the individual’s experience of being a person or ‘self’ is entangled with ‘the quality of … internal experiences of others’ experiences of them’ (Lewis, 2011, location 692). This means that at this order of mind one does not ‘have the capacity to stand apart from the values, beliefs, expectations, or definitions of one’s tribe, community, or culture and make independent judgments about them’ (Kegan, 1998, p. 201).

Individuals who make meaning with a self-authoring mind have distanced themselves from the sense of being entangled in others’ feelings and ideas about themselves. They now have the capacity to be in charge of their own feelings and generate an internal personal meaning system, theory or ideology. Thus, one is able to take as an object the values, beliefs and expectations of others (one’s ‘tribe’, local community, or culture) that one was subject to earlier. Individuals making meaning with this order of mind perceive others as independent entities, with their own integrity, distinct from themselves. Unlike individuals at the socialized order of mind who may struggle heavily with conflicting internalized views, the self-authoring mind tolerates such conflicts or resolves these by invoking a system of self-authored values and knowledge. This system has typically developed over a period of years, gradually integrating the experiences and reflections of personal encounters with a wide variety of other knowledge and value systems (Kegan, 1994). This system of ‘self’ requires strong boundaries, which may prevent the person from recognizing the constructed nature of the system itself. When meeting this construction of self, others may experience it as a somewhat distant way of being, an obstacle to gaining direct contact. However, ‘[t]his greater psychological independence does not mean that [the person is] any less committed to you and to … other close relationships’ (Lewis, 2011, location 1111).

Those individuals who make meaning according to the self-transforming mind have gained a perspective of their own identity construction, and are no longer ‘blind’ to their self-authored identity. At this order, the construction of identity is object to them. This implies that they are now hesitant to see personhood as coinciding with ‘a single system or form’ (Kegan, 1994, p. 313), but rather see their system of self as incomplete and in continuous development. At this order, individuals view the ‘other as part of oneself’ (Souvaine *et al.*, 1990, p. 253) and they are characterized by their embeddedness in a multisystem perspective (Rosen, 1991). These individuals are less likely to view the world in dichotomies, and ‘suspicious of their own tendency to feel wholly identified with one side of any opposite and to identify the other with the other side of that opposite’ (Kegan, 1994, pp. 311-312). Meaning-making with this order of mind concerns the reflections on the process of making meaning itself more than the outcomes of this process. The individual reflects on his or her own need for meaning while acknowledging that knowledge is always partial, and he or she thrives on ‘rending every new veil that comes into awareness, because … closure and fixed boundaries [are] restrictive’ (Cook-Greuter, 1999, p. 107).

In his book *In over our heads: The mental demands of modern life*, Kegan (1994) asks whether people make meaning in accordance with society’s demands. In other words, he asks what order of mind is required to successfully parent, partner, work, learn, heal, and collaborate as modern society frames these life tasks. He shows that society implicitly demands a self-authoring mind for all these tasks. In a composite study sample of adults (Kegan, 1994, p. 195), about half of the persons did not construct their experiences as complexly as the self-authoring mind.

What are the mental demands on ageing in our modern Western society? Does the ageing population meet these demands? Currently, hardly any empirical research exists that answers these questions. Newhouse (as referenced in Kegan, 1998) suggests a number of tasks and expectations typical of the third age: giving up a central identity formed around work and a career, changing from a highly structured to a less structured everyday life, needing to create new friendships after the loss of a ready-made social network, and remaining relatively independent of the care-taking resources of family or society. Kegan infers from Newhouse’s list that it is ‘*the self-authoring mind* that constitutes the implicit mental threshold for successfully handling this curriculum, a threshold many adults will not yet have reached in old age, and not having done so, will be ‘at risk’ for poorer outcomes thereby’ (1998, p. 209; italics in original). Therefore, he argues that it may be ‘an absolutely crucial educational or mental health goal serving as a protective factor against decline and depression in old age’ (Kegan, 1998, p. 212) to develop a self-authoring mind since it is with this order of mind that one can meet the demands of ageing. Moreover, if it is true that more people make meaning with a self-authoring mind, then the social institutions relevant to the third age are challenged to provide the space for the personal paths and demands that are so typical for individuals with this order of mind.

It is against the backdrop of Kegan’s theory and its possible implications for the third age that we now turn to outlining the research we envision. In the following section, we juxtapose the promotion of healthy lifestyles during the third age with Kegan’s psychological development theory.

## DEVELOPING HEALTH PROMOTION FOR THIRD AGERS

Structural developmental theory has informed classroom practice in educational psychology, where developmentally conscious teachers are teaching in ways that encourage students to make meaning in an increasingly complex way, while also meeting students at their stage of development (Helsing *et al.*, 2004). In the context of business coaching and counselling, Berger (2012) refers to this practice as keeping conversations ‘psychologically spacious’. Inspired by such thinking, we envision health promotion initiatives to be psychologically spacious and tailored to a person’s order of mind. Neither our aim nor our interest is in highlighting or facilitating the development towards one specific order of mind (e.g. self-authoring). Our contribution is rather to raise awareness of the qualitatively different ways of making meaning in the world, and, where possible, outline how health promotion can be formulated in developmentally spacious ways, to enable more people to be reached and feel included.

In order to do so, we require a knowledge base that links a person’s lifestyle to his or her stage of structural development. Our research will hopefully help to establish this knowledge base. The rationale for our research is that much information can be gained from the experiences of individuals who report that they have recently transitioned successfully into the third age. In other words, our preferred starting point is narratives concerning a successful lifestyle during retirement, i.e. one that leads to an experience of well-being. True to this experience-oriented bottom-up approach, we employ no specific definition of well-being. The next logical step in our rationale is to relate these situation-specific experiences to a person’s order of mind. Kegan’s measure of order of mind indicates in general terms how a person structures his or her life in terms of responsibility allocation and perspective taking, that is, how a person understands him- or herself to play a role in his or her own life. The assumption is that persons with different orders of mind structure retirement-specific experiences in different ways, because lifestyle and the ensuing experience of well-being are dependent upon order of mind.

More concretely, our research will unfold as follows. We will recruit participants recently retired from working life and reporting having done so satisfactorily according to their own expectations and standards. To assess the participants’ orders of mind, we will conduct subject-object interviews (SOI) (Lahey *et al.*, 1988/2011) with all our participants. During the SOI, ten emotionally laden probes (e.g. ‘Can you tell me of a recent experience of being quite angry about something?’) are presented to a participant, and he or she is asked to write down recent experiences brought to mind by the probes. The participant then selects some of the experiences to elaborate on. During the interview, the interviewer listens sympathetically and confirms the content of the participant’s experience, while also probing for the structuring of the experience. The combination of the emotionally laden probes and the why-questions invites the participants to describe their experiences at the borderline between what is and is not explicitly reflected upon. An analysis of transcripts from the interview allows the researcher to score where participants are on their developmental journey according to Kegan’s developmental theory (1982, 1994). This score indicates whether the participants are currently at one order of mind or in transition between two orders of mind, where four sub-stages can be distinguished. The inter-rater reliability for the SOI ranges between 0.82 to 1.00 for agreement within one discrimination unit (Kegan, 1994; Lahey *et al.*, 1988/2011). We have completed training in subject-object interviewing, are experienced and reliable scorers, and we will establish and report on our inter-rater reliability within this study. If a participant scores at a transitional order of mind, we will allocate him or her according to the dominant order. We are interested to include all adult orders of mind in this study, preferably three participants within each order. However, we are aware of the difficulty of recruiting persons who make meaning at the self-transforming mind as they are few and far between (Kegan, 1994). Knowing this, and given the resources necessary to conduct and analyse such SOIs, it is unlikely that we will be able to recruit enough participants at the self-transforming mind. It is likely that we can include at least three persons at the socialized mind and three at the self-authoring mind, as these are the two orders where most of the adult population makes meaning (Kegan, 1994).

We will conduct an in-depth phenomenological interview with each of the participants. This form of the open qualitative interview will allow us to reveal the phenomenon of well-being as it emerges in the participants’ descriptions of their experiences of the phenomenon (Giorgi, 2009). We have found that three such interviews suffice to make valid inferences about the participants’ experiences with the phenomenon under investigation. That is mainly because a descriptive phenomenological analysis makes use of all data material and is not guided by themes that are established beforehand. We will analyse the descriptions separately for each of the orders of mind, resulting in so-called general meaning structures. Such a general meaning structure reveals the shared meaning across many variations of how participants experience the phenomenon in their daily life (Giorgi, 2009). In a final analysis, we will compare and discuss differences and similarities in the general meaning structure of the phenomenon between the orders of mind. The results of this will feed into the next stage of the project.

## SHAPING STRUCTURAL DEVELOPMENTAL HEALTH PROMOTION

We referred earlier to a quote that a hallmark of health promotion is the aim to bring ‘more life to years, not just years to life’ (Vaillant, 2004, p. 561). One way to bring more life to years is to facilitate experiences of well-being through the promotion of a lifestyle pervaded by such experiences. We will endeavour to make our research results accessible to retirees as well as to the policy-makers and welfare and health promotion professionals who are engaged in their well-being. What do we expect to be able to tell them? What does our research underscore or explicate? In the following, we present a preliminary sketch along three lines of the contribution value of the rationale presented above.

First, both forms of interview will most likely provide information about the shift from working life to retirement. The phenomenological interview aims to capture the general meaning structure of well-being during early retirement. The SOI explores how the individual structures some of his or her recent experiences with change, success, feeling torn, etc. A change of lifestyle that comes with a major shift (such as retiring) appears in the light of a structural developmental approach as either solving a technical problem or overcoming an adaptive challenge (Heifetz and Linsky, 2002). The latter implies a change in order of mind, whereas the former means that the person maintains the same order of mind while incorporating new activities in his or her daily life. For instance, the third age could be lived so that time is increasingly spent on previously well-established activities, or it could incorporate new activities that facilitate or emerge with the structural development of mind. An awareness of the differences between these changes assists the retiree, welfare professional and policy-maker alike in choosing or recommending one activity in favour of another.

Second, both types of interview will provide information about how well-being takes shape in different orders of mind. Following Labouvie-Vief *et al.* (1989), Noam, Young, and Jilnina (2006) have argued that people at various levels of mental complexity may experience and understand their well-being in qualitatively different ways. Bauer (2011) researched the content of the growth stories told by persons with late stages of mental growth (with what he refers to as ‘postconventional selves’). He found that, on average, later stages of development do not necessarily make a person more happy as measured by established quantitative measures of well-being (Diener *et al.*, 1985), which is consistent with Kegan’s theoretical assumptions. One finding, however, stands out, namely that the individuals with the highest score of mental complexity had indeed higher levels of well-being on average when compared to the other stages (Bauer *et al.*, 2011). However, Bauer *et al.* (2011) findings are preliminary, given the relatively small number of participants who scored in the highest stage. Mental complexity, Bauer and colleagues confirm, taps into different aspects of well-being, but their research is inconclusive as to how the first-person experience of well-being relates to mental growth, especially concerning individuals who have not reached the very late stages of development, i.e. the majority of the population.

Kegan (1982, pp. 267-268) has looked into what can be called psychological ‘ill-being’ and its relation to mental complexity. He analysed patient journals at a psychiatric hospital and inferred three different kinds of depression, characterized by three types of loss, respectively: a loss of one’s own needs or the increasing costs of trying to satisfy these needs, a loss of an interpersonal relationship leading to loneliness or even a loss of parts of oneself, and loss of control over meeting one’s own standards. Upon first measuring mental complexity and then relating it to these three types of depression, a strong association between type of depression and mental complexity was observed.

We aim to follow up on the interest of Noam *et al.* (2006) and Bauer et al. in the link between mental complexity and well-being, and use a research design inspired by Kegan’s study of depression. Here we will first divide our participants up into groups according to their SOI score, and then interview them to discover how they experience well-being.

Third, the combination of both interviews will provide essential information to suggest new opportunities for tailoring interventions to the intermediate range between the unique individual and larger cohorts of the population. Tailored interventions have been defined as follows: ‘Any combination of information or change strategies *intended to reach one specific person*, based on characteristics that are unique to that person, related to the outcome of interest, and have been *derived from an individual assessment*’ (Kreuter and Skinner, 2000, p. 1; italics in original). For our purposes, this may be an unattainable ideal considering the amount of resources required. At the other end of the continuum, health promotion that is specific to cohorts, though economically more manageable, may risk not reaching all the members of the targeted population. Consequently, we prefer an intermediate range at which to target the population of retirees. In other words, understanding how individuals with different orders of mind experience well-being differently allows programme developers to tailor psychologically spacious programmes while avoiding individual time-consuming assessments. Moreover, health care and welfare professionals will benefit from an awareness of structural development, lest they under- or overshoot their communication with the target population concerning health promotion activities. Therefore, our research may also help to provide these professionals with knowledge of lifelong development and learning as well as active ageing.

## CONCLUSION

In this article we have outlined perspectives which have as yet not been combined. We have emphasized the notion of adding more life to years as well as the potential for structural developmental thinking in health promotion initiatives. This is an area largely untouched in the health promotion literature, and we see its inclusion as a contribution to extending the positive period of the third age while also aiding the compression of the fourth age.

We have underscored the reciprocity of well-being and lifestyle and have argued that the experience of well-being may have quite different manifestations for different persons when seen through the lenses of a structural development approach. We have sketched a feasible mid-range approach to tailoring health promotion initiatives. This approach attends to the orders of the mind within the target group and has the potential to overcome the practical difficulties of developing unique individual health promotion initiatives.

We have presented one structural developmental theory within the neo-Piagetian tradition as a contrasting view to the current phase theories employed in ageing research, but there are many others which we have not discussed. Notable examples of others in this tradition are Kohlberg (1969), Fowler (1981), Commons *et al.* (1998), Gilligan (1982), Basseches and Mascolo (2009), Cook-Greuter (1999) and Loevinger and Blasi (1976). Kegan’s theory of adult development in health promotion serves our purpose well, which is why we have not focused on other potentially appropriate theories of adult development or mental growth. We conclude that a sensitivity towards the complexity of mind with respect to the experience of well-being will provide healthcare professionals and policy-makers with a powerful tool in their health promotion toolbox.
